# Composition-Driven Structural Regulation of Fe-Co Aerogels for High-Current-Density Hydrogen Evolution

**DOI:** 10.3390/gels12100932

**Published:** 2026-10-16

**Authors:** Sang-Hyun Kim, Haryeong Choi, Younghun Kim, Taehee Kim, Hyung-Ho Park

**Affiliations:** 1 Department of Materials Science and Engineering, Yonsei University, 50 Yonsei-ro, Seodaemun-gu, Seoul 03722, Republic of Korea; sanghyun_kim@yonsei.ac.kr (S.-H.K.); haryeong@yonsei.ac.kr (H.C.); 2 Center for Hydrogen and Fuel Cells, Korea Institute of Science and Technology (KIST), 5 Hwarang-ro 14-gil, Seongbuk-gu, Seoul 02792, Republic of Korea; younghun_kim@kist.re.kr; 3 Department of Materials Science and Engineering, Dankook University, 119 Dandae-ro, Dongnam-gu, Cheonan 31116, Republic of Korea; 4 Aerogel Materials Research Center, Yonsei University, 50 Yonsei-ro, Seodaemun-gu, Seoul 03722, Republic of Korea

**Keywords:** Fe-Co aerogel, hydrogen evolution reaction, poorly crystalline electrocatalyst, alkaline water electrolysis, high-current-density HER, electrochemical accessibility

## Abstract

Cost-effective alkaline water electrolysis requires non-precious-metal electrocatalysts that sustain the hydrogen evolution reaction (HER) at high current densities while maintaining favorable interfacial kinetics and an accessible electrode structure. Here, Co incorporation was used to suppress the long-range crystalline order of Fe and generate poorly crystalline Fe-Co aerogels through one-pot NaBH_4_ reduction, gelation, and freeze-drying. A Fe_100−x_Co_x_ series (x = 0, 60, 80, and 100; nominal feed compositions) was examined to determine how composition-induced structural changes affect network morphology, interfacial accessibility, and high-current HER behavior. Among the series, FeCo60-A formed the finest interconnected particle network and exhibited the largest BET surface area (34.1 m^2^ g^−1^), a high double-layer capacitance (1.04 mF cm^−2^), and the smallest apparent Nyquist arc. After iR correction, it required overpotentials of 345, 520, and 685 mV to reach 100, 500, and 1000 mA cm^−2^, respectively, and maintained a nearly constant potential during 24 h operation at −1000 mA cm^−2^ while largely retaining its interconnected porous morphology. These results demonstrate that Co incorporation into the Fe-based aerogel framework effectively tunes structural order and interfacial characteristics, enabling improved HER performance under high-current alkaline conditions.

## 1. Introduction

Electrochemical water splitting is a central route for coupling renewable electricity to hydrogen production, but catalyst cost, voltage efficiency, and durability at application-relevant current densities remain major constraints. Conventional screening commonly emphasizes 10 mA cm^−2^, whereas application-oriented alkaline electrodes require currents in the hundreds of milliamperes per square centimeter [1,2,3,4,5,6]. Recent oxide-based bifunctional catalysts provide a complementary context: hierarchical CuMn_2_O_4_ and oxygen-vacancy-enriched AgNbO_3_ have shown activity for both alkaline HER and OER, while RbSbWO_6_ has been validated for both reactions in acidic media through reaction-dependent surface states [7,8,9]. Metal-rich aerogel networks are attractive in this context because their fused nanoscale building blocks form a macroscopic, percolated body that can combine a large reactive interface, continuous electron pathways, electrolyte access, and mechanical integrity. The high-current capability of metallic aerogel architectures has been demonstrated by a mechanically robust NiFe aerogel that sustained 500 mA cm^−2^ during oxygen evolution [10]. Recent OER studies on transition-metal aerogels have used low-Ru Fe-Co (oxy)hydroxide matrices, Mn-doped FeNi core–shell networks, and Ni-based bimetallic compositions to tune catalytic sites and their local environment [11,12,13]. Complementary work on self-standing NiFe alloy aerogels and amorphous Fe-Co-La aerogels has highlighted high-current gas management and composition-dependent local coordination, respectively [14,15]. Metal aerogels form through reduction-driven colloidal assembly rather than the molecular condensation mechanism of conventional oxide sol–gels. Metal precursors are reduced to metal-rich nanoparticles, which lose colloidal stability, collide, fuse, and percolate into a self-supporting wet gel; solvent removal then preserves the network as an aerogel [16,17,18,19,20,21,22]. NaBH_4_ can influence both reduction and gelation through its concentration, ionic by-products, and in situ gas evolution. Related routes have produced non-noble Ni-Mo and CoCu aerogels, showing that related reduction-based routes can be extended to non-noble bimetallic systems [23,24]. Structural order is an important composition-dependent variable in multimetal catalysts. Poorly ordered materials can contain diverse coordination environments and defect-rich interfaces [25]. Surface amorphization has also been shown to promote alkaline HER when accompanied by favorable surface chemistry and transport characteristics [26]. These findings highlight the coupled contributions of suppressed long-range order, favorable surface chemistry, and a connected, electrochemically accessible pore network. Composition provides a direct route to tune these coupled structural and electrochemical attributes. Multimetal aerogels can stabilize compositionally mixed, high-area networks, and NaBH_4_-derived Fe-Co aerogels exhibit pronounced Fe/Co-ratio-dependent changes in structural order [27]. Fe-Co alloy catalysts have also been associated with enhanced charge transfer and favorable hydrogen-adsorption energetics in alkaline HER [28,29,30]. Related Ni-Co selenide and Ru-Ni alloy aerogels have also shown favorable electrocatalytic water-splitting behavior [31,32,33,34]. These findings motivate a systematic comparison of Fe-Co aerogels across morphology, pore structure, electrochemical accessibility, and high-current HER performance. In this study, Fe was selected as the primary framework, while Co was introduced to regulate structural order and catalytic performance. Co incorporation suppressed the long-range crystalline order of the Fe-based aerogel and produced poorly crystalline Fe-Co networks. Fe_100−x_Co_x_ aerogels with x = 0, 60, 80, and 100 were synthesized and denoted Fe-A, FeCo60-A, FeCo80-A, and Co-A, respectively. Among these compositions, FeCo60-A exhibited the best overall HER performance, with the lowest Tafel slope and the lowest overpotentials at 100, 500, and 1000 mA cm^−2^. These results indicate that Co incorporation can regulate the aerogel structure and interfacial electrochemical behavior, enabling improved high-current-density HER performance.

## 2. Results and Discussion

### 2.1. Synthesis and Macroscopic Form of Fe-Co Aerogels

**Figure 1:**
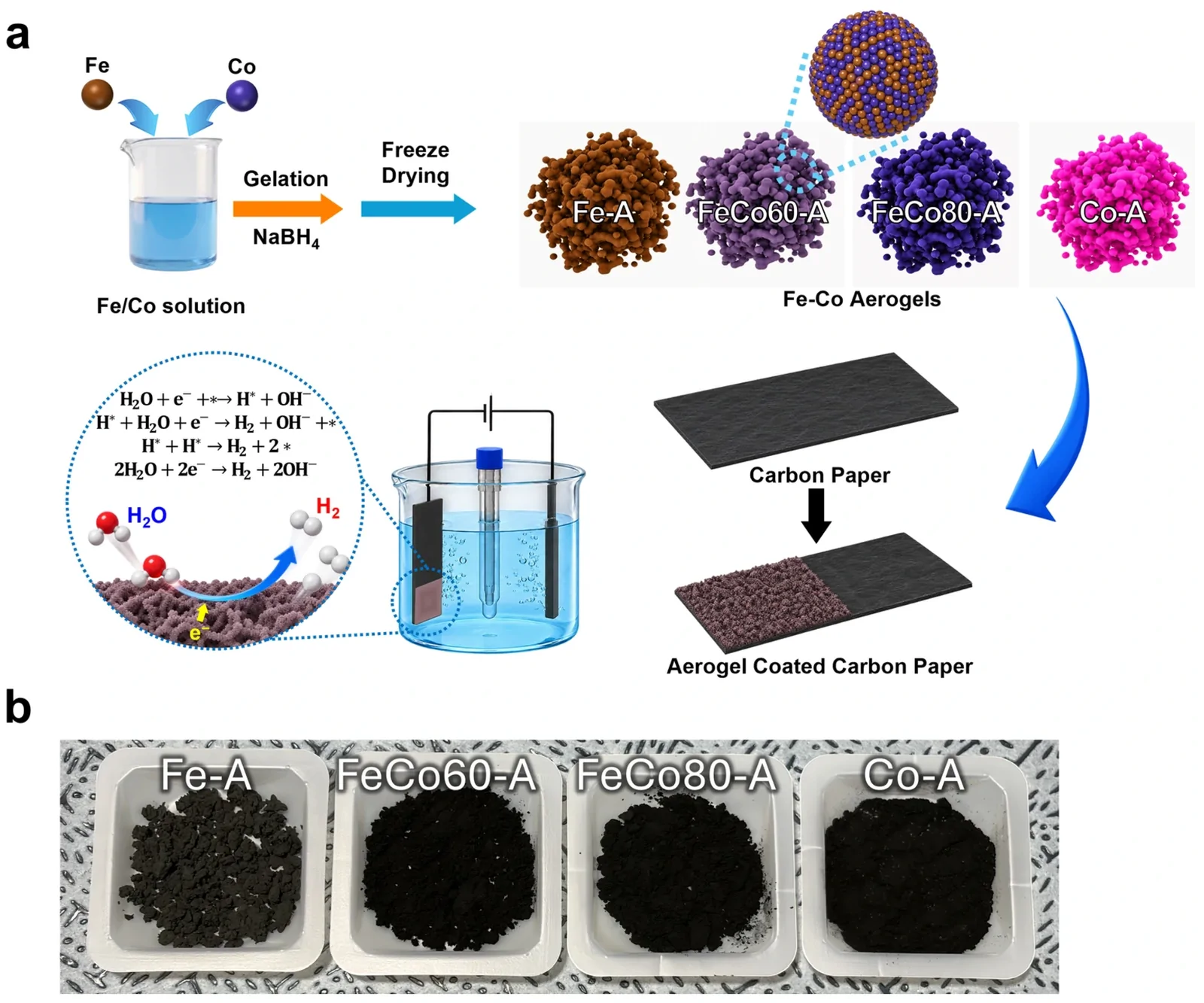
(**a**) Schematic illustration of the synthesis of Fe-Co metal aerogels and the preparation of electrocatalyst electrodes. (**b**) Photographs of the as-synthesized metal aerogels. The asterisk (*) denotes an active site on the catalyst surface, and H* represents an adsorbed hydrogen intermediate.

Figure 1 presents the synthesis route and the resulting macroscopic Fe-Co aerogel products. The process consists of precursor mixing, NaBH_4_-triggered reduction and gelation, freeze-drying, electrode coating, and alkaline HER evaluation.

As illustrated in Figure 1a, Fe and Co precursor solutions were mixed before NaBH_4_ addition, allowing reduction and colloidal assembly to proceed in the same reaction volume. The resulting particle-built wet gel was freeze-dried, processed into a catalyst ink, and coated onto carbon paper for HER evaluation.

Figure 1b shows the dried Fe-A, FeCo60-A, FeCo80-A, and Co-A products. As the nominal Co content increased, their color gradually darkened. After drying, partially fragmented granular aerogels were recovered for all compositions, indicating aerogel formation across the nominal compositional range.

### 2.2. Morphology, Elemental Distribution, and XRD Response

**Figure 2:**
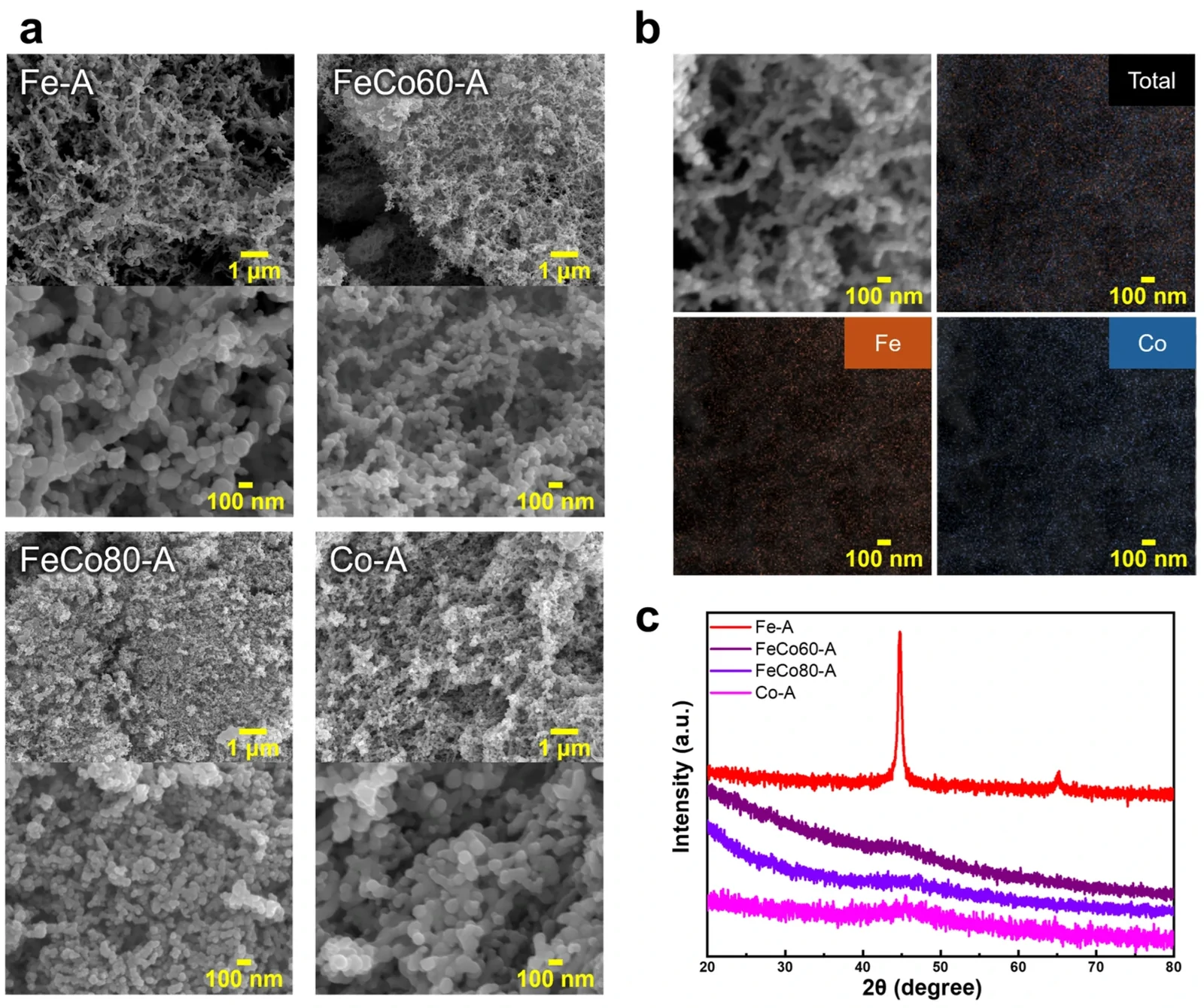
(**a**) SEM images of Fe-A, FeCo60-A, FeCo80-A, and Co-A at different magnifications. (**b**) EDS elemental maps of FeCo60-A showing the Fe and Co signals. (**c**) XRD patterns of Fe-A, FeCo60-A, FeCo80-A, and Co-A.

The SEM images in Figure 2a show interconnected networks assembled from fused nanoscale particles for all compositions. Fe-A contains elongated chains and necked particles that form comparatively open voids. FeCo60-A exhibits a fine, continuous skeleton with abundant junctions at both the 1 μm and 100 nm scales. FeCo80-A appears more granular and locally compact, whereas Co-A contains coarser rounded building blocks. These observations indicate that nominal Co content influences the size and packing of the network-forming units while preserving the interconnected porous architecture.

The FeCo60-A EDS maps in Figure 2b show Fe and Co signals distributed throughout the same mapped region, indicating local elemental mixing within the observed network. The bulk Fe:Co atomic ratios were quantified by inductively coupled plasma optical emission spectroscopy (ICP-OES), as summarized in Table S4. The measured Fe:Co ratios of FeCo60-A and FeCo80-A were 38.44:61.56 and 18.67:81.33, respectively, close to the nominal precursor ratios of 40:60 and 20:80. These results confirm that the resulting aerogels approximately retained the intended Fe–Co compositions. The XRD patterns in Figure 2c reveal a clear composition-dependent change in structural order. Fe-A displays a strong reflection near 44.7° and a weaker feature near 65°, corresponding to the dominant (110) and (200) reflections of bcc Fe. In contrast, FeCo60-A, FeCo80-A, and Co-A exhibit broad, low-intensity diffraction profiles without sharp Fe-derived reflections, indicating reduced long-range crystalline order. The Co-containing aerogels are therefore characterized as poorly crystalline or highly disordered structures, consistent with the composition-dependent disorder reported for related Fe-Co aerogels [18,22,27,35].

### 2.3. Surface Area, Nanoscale Morphology, and Surface Chemical States

**Figure 3:**
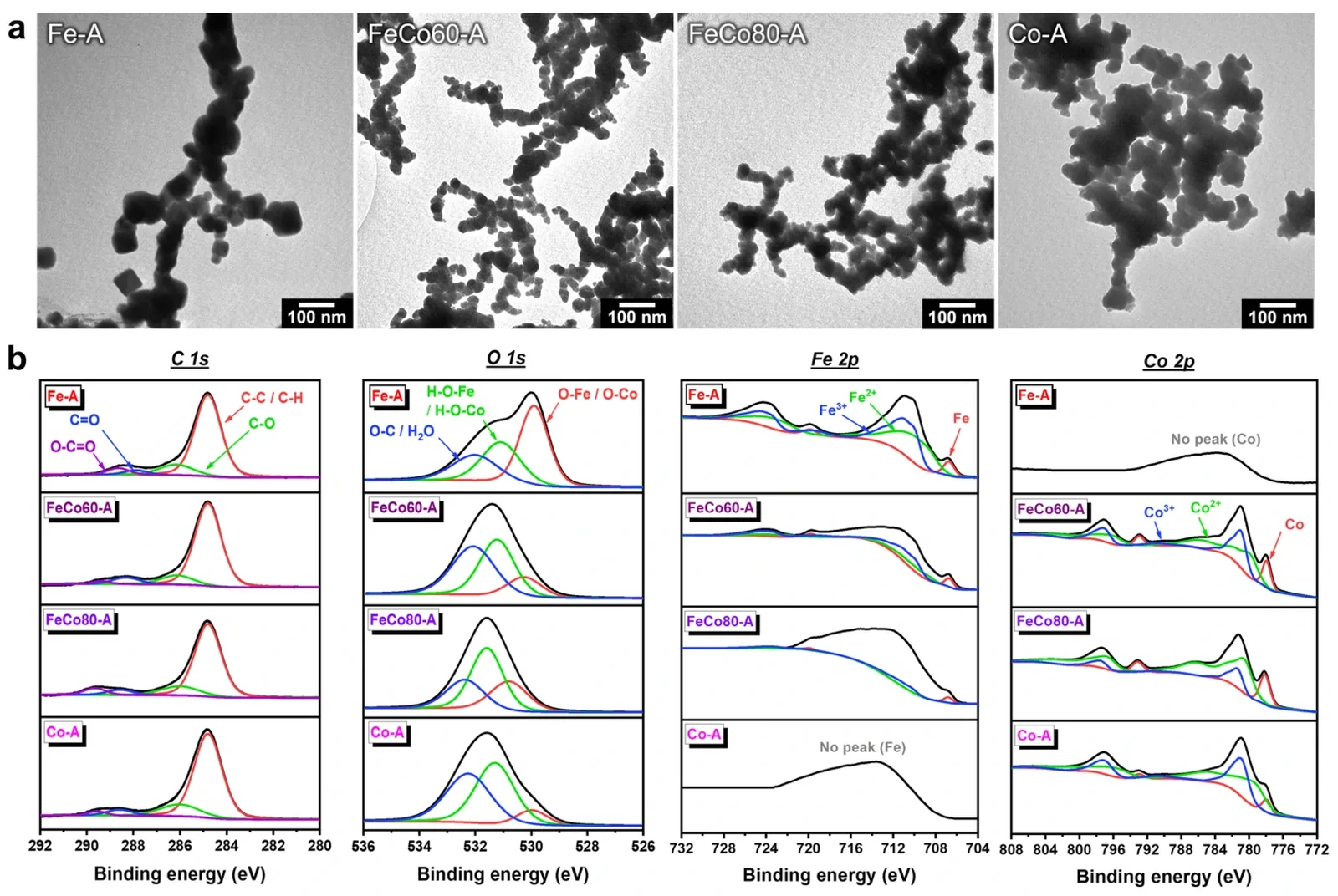
(**a**) TEM images of Fe-A, FeCo60-A, FeCo80-A, and Co-A (scale bars: 100 nm). (**b**) High-resolution XPS spectra of the C 1s, O 1s, Fe 2p, and Co 2p regions for Fe-A, FeCo60-A, FeCo80-A, and Co-A, with peak deconvolution. The peak assignments indicated by the arrows in each XPS region apply to the corresponding fitted components of the other samples.

The TEM images in Figure 3a corroborate the interconnected particle networks observed by SEM (Figure 2a). Fe-A contains relatively coarse, elongated units connected through particle necks, whereas FeCo60-A exhibits a finer branched network with numerous particle junctions. FeCo80-A and Co-A show comparatively coarser or more densely assembled building units. The broad XRD profiles of the Co-containing samples (Figure 2c) suggest reduced long-range crystalline order. Related composition-sensitive diffraction features and reduced crystallinity have also been reported for Fe–Co–La (oxy)hydroxide aerogels and precursor-ratio-controlled iron-oxide aerogels [15,36]. These observations suggest that the aerogels with relatively high Co fractions in the present series possess low crystallinity.

Nitrogen adsorption–desorption measurements complement these morphological observations (Figure S1a–f). The BET surface areas of Fe-A, FeCo60-A, FeCo80-A, and Co-A are 23.3, 34.1, 29.2, and 17.8 m^2^ g^−1^, respectively. Their total pore volumes are 0.046, 0.071, 0.075, and 0.060 cm^3^ g^−1^, and the corresponding average pore diameters are 7.89, 8.37, 10.22, and 13.41 nm. All samples contain broadly distributed mesopores, with an overall increase in average pore diameter toward Co-rich compositions. FeCo60-A combines the largest surface area with an intermediate pore diameter and pore volume. Together with SEM and TEM, this result indicates that its finer network exposes a larger nitrogen-accessible interface.

The C 1s spectra in Figure 3b retain a dominant C–C/C–H component near 284.8 eV throughout the composition series. The smaller C–O, C=O, and O–C=O contributions remain secondary as Fe-A is replaced by FeCo60-A and then by the more Co-rich samples. This broadly similar carbon envelope is consistent with adventitious carbon containing a major aliphatic component and smaller oxygenated contributions [37,38].

The Fe 2p and Co 2p spectra reveal a more pronounced compositional response. Fe-A displays distinct oxidized-Fe features in the approximately 709–713 eV region together with a smaller metallic-Fe component near 706–707 eV. In FeCo60-A, the oxidized-Fe features become less pronounced, while the metallic-Fe component remains visible and Co^0^, Co^2+^, and Co^3+^ contributions appear in the Co 2p spectrum. Upon further Co enrichment to FeCo80-A, the resolved Fe-related features weaken further, consistent with its lower Fe content, whereas the Co^2+^-assigned envelope becomes more prominent relative to the Co^3+^-assigned contribution. Co-A retains a pronounced oxidized-Co envelope and a less distinct metallic-Co contribution than the binary aerogels. These changes suggest that introducing Co reorganizes the distribution of metallic and oxygen-coordinated environments, with FeCo60-A retaining both Fe- and Co-containing surface species. The coexistence of metallic and oxidized components is consistent with the surface-state assignments reported for Fe and Co [37]. A similar coupling between Fe–Co electronic interaction, mixed surface chemical states, and improved alkaline HER has been reported for a Prussian blue analog-derived FeCo alloy [28].

The O 1s spectra show how this redistribution affects oxygen coordination. In Fe-A, the low-binding-energy metal–oxygen component near 530 eV is dominant. Upon moving to FeCo60-A, this component becomes much smaller relative to the metal–OH contribution near 531–532 eV and the higher-binding-energy contribution associated with carbon-bound oxygen and adsorbed water. The change suggests a transition from an oxide-dominated oxygen environment toward a more hydroxylated and hydrated surface. From FeCo60-A to FeCo80-A, the metal–OH component becomes more prominent relative to the higher-binding-energy contribution, and the metal–oxygen component becomes more visible again. Co-A also exhibits a predominantly hydroxyl-containing envelope with a relatively small low-binding-energy metal–oxygen contribution. These two compositional transitions highlight the sensitivity of metal–O, metal–OH, and adsorbed oxygen-containing environments to the Fe–Co ratio [37]. Such a balance may influence water adsorption, dissociation, and OH removal at adjacent metal sites; hydroxylated FeCo surfaces have been reported to balance water activation and OH removal during alkaline HER [39].

Taken together, these trends suggest that FeCo60-A combines a fine, poorly crystalline network with metallic Fe–Co environments and a hydroxylated surface capable of interacting with water. Further Co enrichment produces a coarser network, a smaller BET surface area, and a redistribution toward more prominent metal–OH contributions while reducing the availability of Fe-containing environments. This combination may alter the balance between water activation and hydrogen adsorption and provides a structural and chemical basis for the composition-dependent HER response discussed below.

### 2.4. High-Current HER Activity and Apparent Interfacial Kinetics

**Figure 4:**
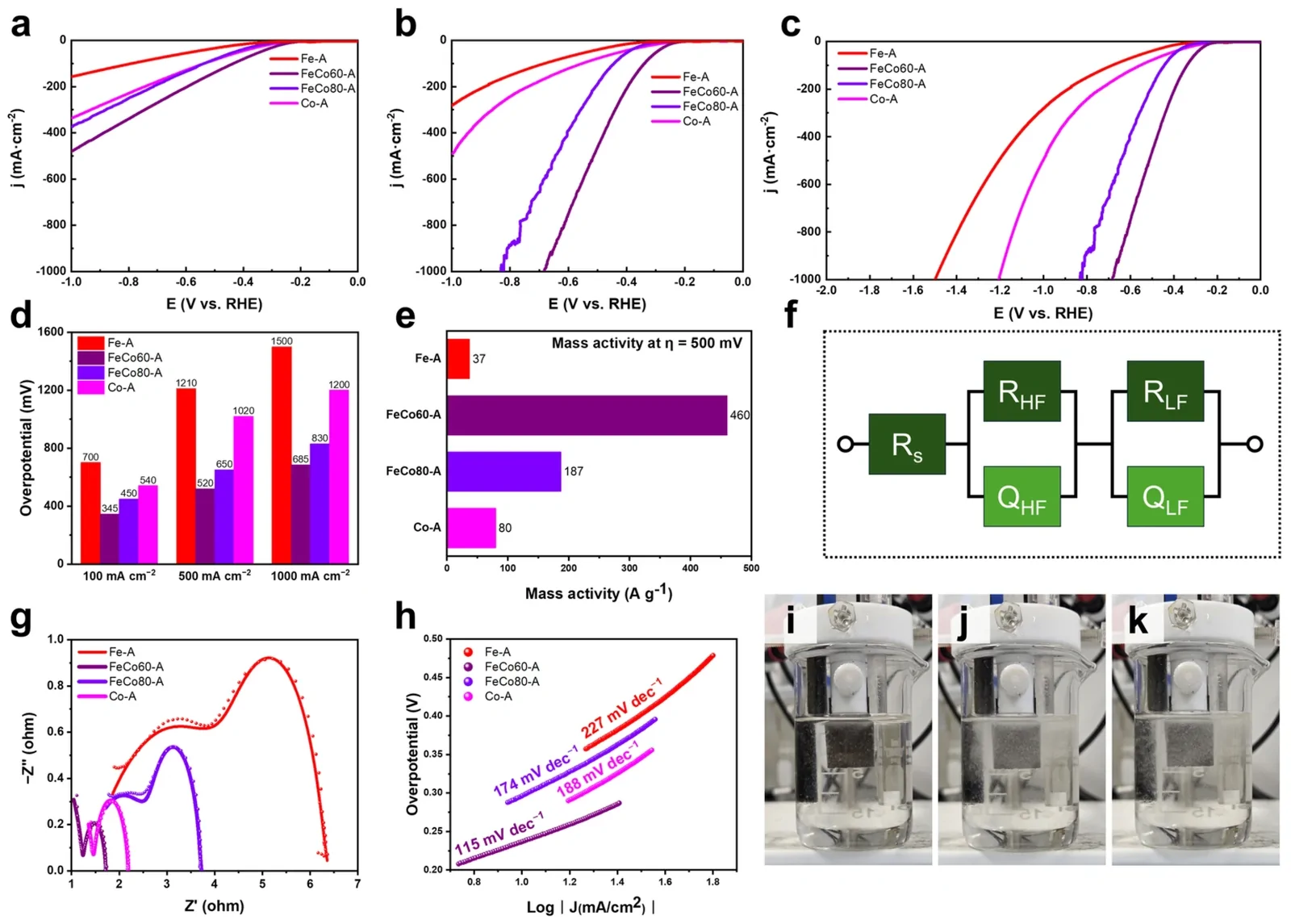
HER electrocatalytic performance of Fe-Co metal aerogels. (**a**) Uncorrected and (**b**,**c**) iR-corrected HER polarization curves of Fe-A, FeCo60-A, FeCo80-A, and Co-A. (**d**) Overpotentials at 100, 500, and 1000 mA cm^−2^. (**e**) Mass activity at η = 500 mV. (**f**) Equivalent circuit model for EIS fitting and (**g**) Nyquist plots with the corresponding equivalent-circuit fits. Symbols represent the experimental data, and solid lines represent the fitted curves. (**h**) Comparison of Tafel slopes. (**i**–**k**) Photographs of HER operation at 100, 500, and 1000 mA cm^−2^, respectively.

Figure 4a–c compare the HER polarization behavior of the four aerogels. Figure 4a shows the uncorrected polarization curves, whereas Figure 4b,c show the iR-corrected curves over different potential ranges. After iR correction, FeCo60-A reaches a given cathodic current density at the least negative potential, followed by FeCo80-A, Co-A, and Fe-A. The overpotential comparison in Figure 4d further shows this activity order. FeCo60-A requires 345, 520, and 685 mV to reach 100, 500, and 1000 mA cm^−2^, respectively. The corresponding values are 700, 1210, and 1500 mV for Fe-A; 450, 650, and 830 mV for FeCo80-A; and 540, 1020, and 1200 mV for Co-A. FeCo60-A therefore exhibits the lowest overpotential at all three tested current densities. The mass-normalized comparison in Figure 4e further highlights the activity of FeCo60-A. At η = 500 mV, FeCo60-A reaches 460 A g^−1^, compared with 37 A g^−1^ for Fe-A, 187 A g^−1^ for FeCo80-A, and 80 A g^−1^ for Co-A. Thus, FeCo60-A exhibits approximately 12.4- and 2.46-fold higher mass activities than Fe-A and FeCo80-A, respectively. In Table S2, A comparison with representative non-precious transition-metal-based HER electrocatalysts in 1 M KOH is summarized. FeCo60-A/CP achieves an overpotential of 685 mV at 1,000 mA cm^−2^ and maintains stable operation at this current density for 24 h, demonstrating its capability for sustained high-current-density HER. The Nyquist plots in Figure 4g were fitted using the equivalent circuit R_s_-(R_HF_ ∥ Q_HF_)-(R_LF_ ∥ Q_LF_). The fitted R_LF_ values were 1.561, 0.474, 0.877, and 0.720 Ω for Fe-A, FeCo60-A, FeCo80-A, and Co-A, respectively. FeCo60-A exhibited the lowest R_LF_, indicating the lowest interfacial resistance among the four electrodes [40]. The corresponding fitting root-mean-square error (RMSE) values were 0.0359, 0.0135, 0.0202, and 0.0147 Ω, respectively, confirming good agreement between the experimental and fitted Nyquist responses (Table S3).

The Tafel plots in Figure 4h give slopes of 227, 115, 174, and 188 mV dec^−1^ for Fe-A, FeCo60-A, FeCo80-A, and Co-A, respectively. FeCo60-A exhibits the lowest-fitted Tafel slope in the selected range, consistent with the most favorable apparent HER kinetics among the four compositions [29,30,41]. Figure 4i–k show representative photographs of FeCo60-A during HER operation at −100, −500, and −1000 mA cm^−2^, respectively. Visible H_2_ bubble evolution was observed at the electrode surface at all three current densities, with more vigorous gas evolution at higher current densities, providing visual evidence of sustained HER operation under high-current-density conditions [6,42,43,44]. Taken together, the polarization, mass-activity, Nyquist, and Tafel results consistently identify FeCo60-A as the most active composition in the tested series. Its enhanced HER performance coincides with the fine interconnected morphology, the highest BET surface area, a relatively high electrochemical accessibility, and the smallest apparent Nyquist arc. The combined structural and electrochemical characteristics of FeCo60-A are therefore correlated with its high-current-density hydrogen evolution performance [18,22,33,34].

### 2.5. Electrochemical Accessibility and Constant-Current Stability

**Figure 5:**
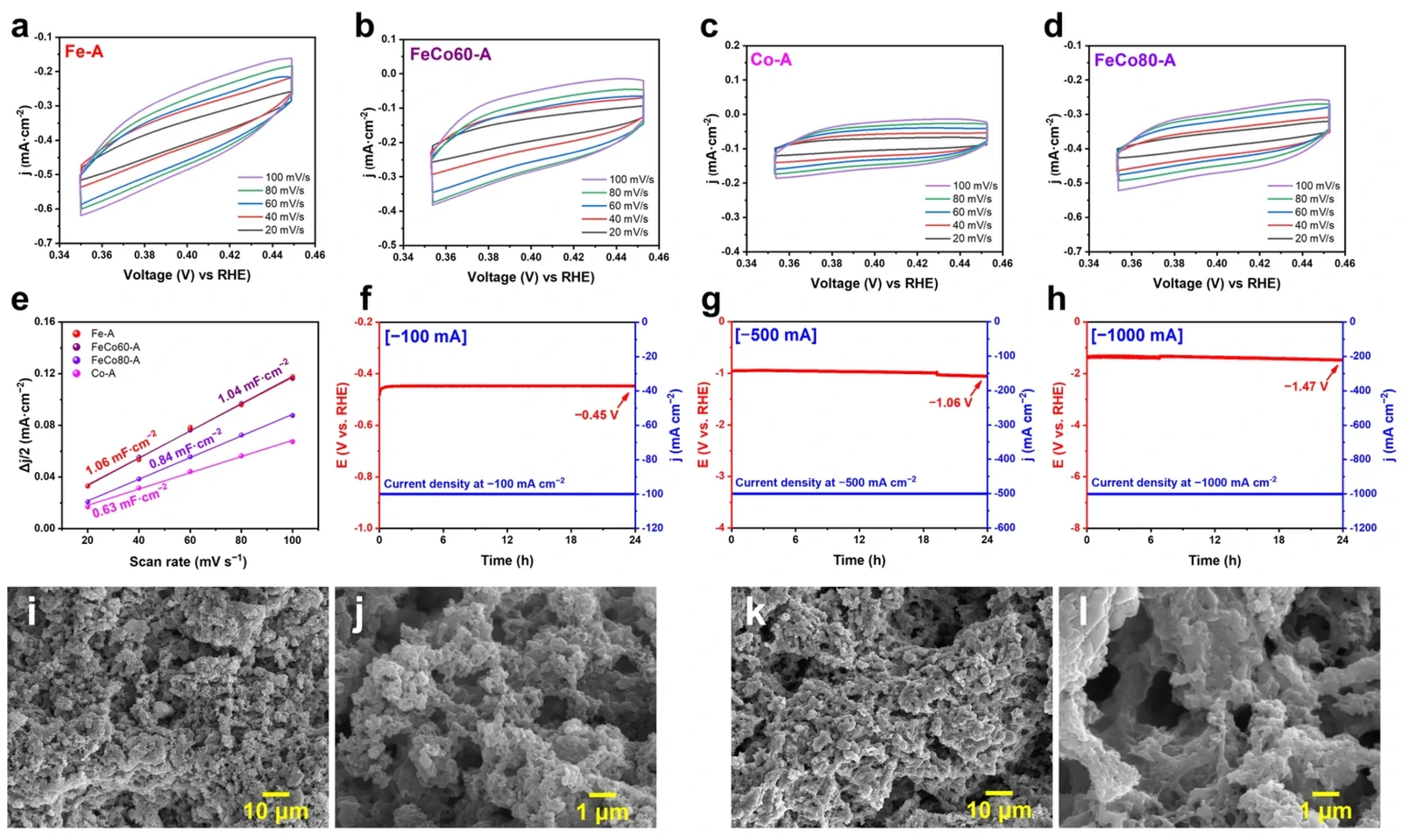
Electrochemical accessibility and constant-current stability of Fe-Co metal aerogels. (**a**–**d**) CV curves at different scan rates for C_dl_ determination. (**e**) C_dl_ plots derived from the CV curves. (**f**–**h**) Galvanostatic chronopotentiometric stability of FeCo60-A at −100, −500, and −1000 mA cm^−2^ for 24 h, respectively. The recorded potentials are shown without iR correction. SEM images (**i**,**j**) before and (**k**,**l**) after the 24 h stability test at −1000 mA cm^−2^.

The CV curves in Figure 5a–d were collected between 0.35 and 0.45 V versus RHE at scan rates of 20–100 mV s^−1^. The slopes of Δj/2 versus scan rate (Figure 5e and Table S1) yielded C_dl_ values of 1.06, 1.04, 0.84, and 0.63 mF cm^−2^ for Fe-A, FeCo60-A, FeCo80-A, and Co-A, respectively. Fe-A and FeCo60-A display the largest charging responses in this series, while increasing the Co fraction beyond FeCo60-A decreases C_dl_. These trends indicate that FeCo60-A maintains a relatively large electrochemically accessible interface within its fine interconnected network.

FeCo60-A combines the finest interconnected network, the highest BET surface area, a relatively high C_dl_, and the most favorable polarization, mass-activity, Tafel, and impedance responses. Fe-A also has a high C_dl_, while the lower overpotentials of FeCo60-A indicate a beneficial contribution from its binary composition and surface chemistry. Likewise, the lower HER activity of FeCo80-A, despite its broad XRD features, emphasizes the favorable combination of network fineness and Fe–Co surface environments at the intermediate composition. The activity maximum of FeCo60-A therefore suggests a coupled contribution from accessible interfaces, heterometallic environments, and oxygen coordination.

Quantitative analysis of reaction energetics requires atomic models representative of the low crystallinity and partially oxidized Fe–Co surface. Reliably comparing reaction energies while accounting for the structural disorder of the present aerogels would require constructing and evaluating multiple local atomic configurations and adsorption sites, involving substantial computational resources. The scale of such sampling is illustrated by an amorphous-alloy HER study that combined machine learning and DFT to evaluate approximately 40,000 candidate active sites [45].

A mechanistic interpretation emerges from related Fe–Co and aerogel catalysts. In a Prussian blue analog-derived FeCo alloy, electronic interaction between Fe and Co modified hydrogen adsorption and accelerated interfacial charge transfer [28]. Composition optimization in a three-dimensional porous FeCo@NC/carbon-nanosheet catalyst likewise coupled finely dispersed FeCo units with mesoporous and macroporous pathways to enhance alkaline HER [46]. A further FeCo study identified a metallic core with an oxide/hydroxide surface and showed that hydroxylated FeCo(110) can balance water activation and OH removal [39]. Related amorphous Fe-, Co-, and Ni-based aerogels also exhibit HER behavior governed by a combination of porous morphology, surface oxygen environments, and the intrinsic properties of the metal centers [47].

For FeCo60-A, the metallic-Fe and Co contributions in XPS suggest sites that can accommodate adsorbed hydrogen, while neighboring metal–OH and oxygen-coordinated environments may facilitate the adsorption and activation of water. Fe–Co electronic coupling may tune hydrogen binding at these sites, and the hydroxylated surroundings may help balance water dissociation and the release of OH-containing intermediates [28,39]. The relatively balanced metal–OH and higher-binding-energy oxygen contributions of FeCo60-A are consistent with an interface that supports both water interaction and hydrogen conversion. Increasing the Co fraction to FeCo80-A makes the metal–OH contribution more prominent while decreasing Fe-containing environments and coarsening the network. These changes may reduce the accessibility of complementary Fe–Co environments and shift intermediate binding away from the favorable balance obtained at FeCo60-A. Its finer network and largest BET area further promote contact with electrolytes and access to particle junctions, consistent with its relatively high C_dl_. The lowest Tafel slope of 115 mV dec^−1^ and fitted low-frequency interfacial resistance of 0.474 Ω support faster interfacial HER processes at this composition. The combined effect of local surface chemistry and network accessibility thus offers a plausible explanation for the superior high-current HER performance of FeCo60-A.

Figure 5f–h show the constant-current response of FeCo60-A at −100, −500, and −1000 mA cm^−2^, respectively. After an initial conditioning period, the potentials approach approximately −0.45, −1.06, and −1.47 V versus RHE and remain nearly steady throughout the 24 h test. These chronopotentiometric potentials were recorded without iR correction and include the uncompensated ohmic voltage drop. Sustained operation at all three current densities, including −1000 mA cm^−2^, indicates that the interconnected catalyst electrode maintains a stable response during high-current hydrogen evolution [43,44,48].

The SEM images in Figure 5i–l show that FeCo60-A retains an interconnected porous framework after 24 h of HER operation at −1000 mA cm^−2^, accompanied by local particle coarsening and enlargement of the voids. ICP-OES analysis of the electrolyte before and after testing showed no detectable Fe or Co (Table S5). Together, these observations suggest that the morphological changes mainly involve rearrangement within the retained aerogel framework.

Ex situ XPS of FeCo60-A/CP before and after the 24 h HER stability test reveals a redistribution of surface chemical states within the retained catalyst framework (Figure S2). The metallic-Fe contribution near 706–707 eV remains evident after HER and becomes more prominent relative to the Fe^2+^/Fe^3+^ envelope near 709–713 eV. This change is consistent with cathodic conditioning that partially reduces oxygen-coordinated Fe or exposes more metallic Fe-containing regions. In Co 2p, the Co^2+^-assigned feature around 780–782 eV becomes narrower after HER, while the higher-binding-energy shoulder around 785–786 eV becomes more apparent and oxidized-Co contributions remain visible. The simultaneous retention of metallic Fe and redistribution of oxidized-Co features suggests preferential adjustment of Fe-containing regions together with reorganization of the oxygen-coordinated Co environment [37]. Related porous FeCo@NC catalysts also retain metallic and oxidized Fe/Co components after HER, accompanied by changes in their relative contributions and partial reduction in oxidized Co [46]. These observations support redox conditioning of a mixed metallic and oxygen-containing Fe–Co surface during prolonged hydrogen evolution.

The O 1s spectrum retains the low-binding-energy metal–O contribution near 530 eV and the metal–OH contribution near 531–532 eV after testing. The approximately 533 eV contribution becomes more prominent relative to metal–OH, while the higher-binding-energy component near 535–536 eV remains observable. The C 1s spectrum simultaneously shows increased prominence of the C–C/C–H component near 284.8 eV relative to the Nafion-derived –CF_2_– component near 292 eV. The smaller C–O/C–F, CF_2_–O, and –CF_3_ contributions also persist. These coordinated changes suggest redistribution of the catalyst–binder–carbon interface as the electrode is wetted and the aerogel network locally rearranges. Nafion has a –CF_2_– C 1s contribution near 292.2 eV and sulfonate and ether O 1s contributions near 533.1 and 535.5 eV, respectively; changes in surface exposure of its polar side-chain groups upon conditioning have also been reported [49]. In the present electrode, greater relative exposure of carbon-paper regions and oxygen-containing binder groups may account for the more evident C–C/C–H signal and the redistribution of the high-binding-energy O 1s envelope.

The retained metallic-Fe signal, oxygen-coordinated Co features, and metal–OH contribution suggest an interface in which metallic environments support hydrogen adsorption while adjacent oxygen-containing environments assist in water interaction. Such complementary functions are consistent with FeCo catalysts whose hydroxylated surfaces balance water activation and OH removal [39]. The post-HER spectra therefore suggest that surface conditioning preserves this combination of functions while adjusting the relative exposure and coordination of the contributing species. Together with the stable potential response, this interpretation supports sustained HER on a chemically reorganized but interconnected Fe–Co interface.

Local coarsening after HER may arise from particle migration and aggregation promoted by prolonged hydrogen evolution and surface conditioning. Increased particle mobility under more cathodic HER conditions has been observed for carbon-supported Pt and linked to hydrogen generation and changes in particle–support interactions [50]. Larger agglomerates composed of fine particles were also observed in a NiMo/KB HER cathode after 2000 h of electrolysis at 1 A cm^−2^, together with the reduction in oxygen-containing surface species [51]. By analogy, repeated bubble growth and detachment, together with an adjustment of surface oxygen coordination, may promote the movement and consolidation of neighboring units in FeCo60-A, producing coarser particles and larger voids. The retained interconnected morphology, limited potential drift, and absence of detectable Fe or Co in the analyzed electrolyte suggest that this local reorganization remains compatible with continuous electronic pathways and electrolyte access during the 24 h test.

## 3. Conclusions

In this study, composition-controlled Fe-Co aerogels were evaluated as non-precious electrocatalysts for high-current-density hydrogen evolution in alkaline media. Co incorporation markedly altered the structural order and network characteristics of the Fe-based aerogels, producing poorly crystalline Fe-Co frameworks with composition-dependent morphology and pore structures. Among the investigated compositions, FeCo60-A exhibited the most favorable overall combination of structural and electrochemical properties, featuring a fine interconnected particle network, the highest BET surface area of 34.1 m^2^ g^−1^, the smallest apparent Nyquist arc, and the lowest Tafel slope of 115 mV dec^−1^. FeCo60-A required overpotentials of 345, 520, and 685 mV to reach 100, 500, and 1000 mA cm^−2^, respectively, and achieved a mass activity of 460 A g^−1^ at an overpotential of 500 mV. The relatively high C_dl_ of FeCo60-A, together with its favorable polarization and interfacial response, suggests that its optimized Fe-Co composition, accessible porous architecture, suppressed long-range structural order, and surface chemical environments jointly promote high-current HER. FeCo60-A further showed limited potential drift during 24 h operation at cathodic current densities up to −1000 mA cm^−2^, while its interconnected porous framework remained visible despite local morphological reconstruction. These findings indicate that a composition-driven control of the structure and the electrochemical interface of Fe-Co aerogels is a promising strategy for developing non-precious electrocatalysts for high-current-density hydrogen evolution.

The composition-dependent XPS features and their evolution after HER suggest that metallic Fe–Co environments and neighboring oxygen-containing species work together at the catalytic interface. At FeCo60-A, this surface chemistry coincides with the finest network, highest BET area, favorable interfacial kinetics, and stable 24 h operation. These correlations support composition control as a means of balancing water activation, hydrogen adsorption, and access to the interconnected catalyst surface.

## 4. Materials and Methods

### 4.1. Materials

Sodium borohydride (NaBH_4_, 98%), cobalt(II) chloride hexahydrate (CoCl_2_·6H_2_O, 98%), iron(III) chloride hexahydrate (FeCl_3_·6H_2_O, 97%), potassium hydroxide (KOH, 85%), and ethanol (99.5%) were purchased from Sigma-Aldrich (St. Louis, MO, USA) and used as received. Nafion solution (5 wt%) was purchased from Chemours (Wilmington, DE, USA) and used as received. Ultrapure deionized (DI) water produced using a laboratory ultrapure-water purification system was used for all aqueous preparations.

### 4.2. Synthesis of the Selected Fe-Co Aerogels

Four nominal feed compositions were selected from the Fe_100−x_Co_x_ series: x = 0, 60, 80, and 100. The resulting aerogels were denoted Fe-A, FeCo60-A, FeCo80-A, and Co-A, respectively, where the number indicates the nominal Co molar proportion and A denotes aerogel. Separate 0.05 M aqueous solutions of FeCl_3_·6H_2_O and CoCl_2_·6H_2_O were prepared using ultrapure DI water. Appropriate volumes of the precursor solutions were combined to obtain nominal Fe:Co molar ratios of 100:0, 40:60, 20:80, and 0:100 while maintaining a total metal concentration of 0.05 M and a total precursor solution volume of 10 mL. Subsequently, 15 mL of freshly prepared 0.10 M NaBH_4_ aqueous solution was introduced into the metal-salt precursor solution at an addition rate of approximately 15 mL s^−1^ under static conditions without external stirring. The amount of NaBH_4_ corresponded to a NaBH_4_-to-total-metal molar ratio of 3:1. Rapid reduction produced black products, and gel formation proceeded as the initially formed nanoparticles progressively aggregated. Gelation was considered complete when the surrounding solution became visually transparent, which occurred within approximately 2–6 h depending on the composition, with longer gelation times observed at higher Co contents. The resulting wet gels were then aged for 6 h. After aging, the gels were repeatedly washed with ultrapure DI water approximately 5–6 times until the washing solution became clear and colorless to remove soluble by-products. The washed wet gels were rapidly frozen using liquid nitrogen and subsequently lyophilized for 48 h under a pressure of 0.050 Torr, with the condenser maintained at −150 °C, to obtain the Fe-Co aerogels.

### 4.3. Materials Characterization

The morphology and elemental distribution of the Fe-Co aerogels were examined by scanning electron microscopy (SEM, JSM-7001F, JEOL Ltd., Tokyo, Japan) and energy-dispersive X-ray spectroscopy (EDS). The Fe and Co contents of the aerogels were determined by inductively coupled plasma optical emission spectroscopy (ICP-OES; Optima 8300, PerkinElmer, Waltham, MA, USA). The Fe:Co atomic ratios were calculated from the measured elemental mass fractions using the respective atomic weights and normalized to the total Fe and Co content. ICP-OES was also used to quantify Fe and Co concentrations in the electrolyte before and after HER stability testing to evaluate potential metal leaching. The structural order was examined by powder X-ray diffraction (XRD, Rigaku Ultima IV, Rigaku Corporation, Tokyo, Japan) using Cu Kα radiation (λ = 1.54 Å) over a 2θ range of 20–80°, with a step size of 0.02° (2θ) and a scan rate of 4° min^−1^. The XPS spectra were deconvoluted using XPSPEAK software (version 4.1, Hong Kong, China). N_2_ adsorption–desorption measurements were performed at 77.35 K using an Autosorb iQ-MP surface area and pore size analyzer (Quantachrome Instruments, Boynton Beach, FL, USA). Prior to the measurements, the samples were degassed at 120 °C under vacuum (~0.01 Torr) for 24 h. The specific surface area was determined using the multipoint Brunauer–Emmett–Teller (BET) method [52,53]. The pore size distribution was calculated using the Barrett–Joyner–Halenda (BJH) method from the desorption branch of the N_2_ isotherm [52,54]. The total pore volume was determined at a relative pressure of P/P_0_ ≈ 0.984, and the average pore diameter (D_avg_) was calculated according to D_avg_ = 4V_total_/S_BET_, where V_total_ and S_BET_ represent the total pore volume and BET specific surface area, respectively.

### 4.4. Electrochemical Measurements

Electrochemical measurements were conducted using a ZIVE electrochemical workstation (WonATech Co., Ltd., Seoul, Republic of Korea). The working electrodes were prepared by coating Fe-A, FeCo60-A, FeCo80-A, and Co-A onto carbon paper (CP). The catalyst ink was prepared by dispersing the fabricated aerogel and a 5 wt% Nafion solution in a 1:1 (*v*/*v*) ethanol/water mixture at a catalyst-to-Nafion-solution mass ratio of 1:35. The resulting ink was then deposited directly onto carbon paper (CP). A graphite electrode and an Hg/HgO electrode (RE-61AP, BAS Inc., Tokyo, Japan, 1 M NaOH internal electrolyte) were used as the counter and reference electrodes, respectively, in a three-electrode configuration. The electrolyte was 1 M aqueous KOH, and the electrochemical measurements were conducted at room temperature (approximately 25 °C). The coated electrode area was 1.0 cm^2^, and the catalyst loading was approximately 1 mg cm^−2^. Potentials were converted to the reversible hydrogen electrode (RHE) scale using E(RHE) = E(Hg/HgO) + 0.9688 V. The conversion factor was calculated according to E(RHE) = E(Hg/HgO) + E_ref_(Hg/HgO) + 0.0592 × pH at 25 °C, using an Hg/HgO reference potential of 0.140 V for the 1 M NaOH internal electrolyte and an assumed pH of 14 for 1 M KOH, yielding a conversion offset of 0.9688 V. Linear sweep voltammetry was recorded at 5 mV s^−1^ in quiescent electrolyte without rotation or mechanical stirring. The uncompensated solution resistance (R_s_) for each electrode was determined from the high-frequency region of the corresponding Nyquist plot and was used for iR correction of the polarization curves. Current density was normalized to the exposed geometric area according to j = I/A; because the exposed area was 1.0 cm^2^, the numerical values of current (mA) and geometric current density (mA cm^−2^) are identical. Potentiostatic EIS measurements were performed for all samples at −1.40 V versus Hg/HgO using a 10 mV AC perturbation over the frequency range from 100 kHz to 0.10 Hz. A logarithmic frequency sweep with 10 points per decade was applied. The Nyquist spectra were fitted using ZMAN software 2.4 (WonATech Co., Ltd., Seoul, Republic of Korea) with an R_s_-(R_HF_ ∥ Q_HF_)-(R_LF_ ∥ Q_LF_) equivalent circuit, where R_s_ represents the solution resistance, R_HF_ and R_LF_ represent the resistance components associated with the high- and low-frequency interfacial processes, respectively, and Q_HF_ and Q_LF_ are the corresponding constant-phase elements. For C_dl_ determination, cyclic voltammograms were collected from 0.35 to 0.45 V versus RHE at scan rates of 20, 40, 60, 80, and 100 mV s^−1^. Non-Faradaic CV measurements were repeated using higher-resolution current and potential acquisition settings to improve the measurement of small charging signals. The anodic and cathodic current densities, j_a_ and j_c_, respectively, were extracted at 0.40 V versus RHE, and the current separation was calculated as Δj/2 = (j_a_ − j_c_)/2. The slope of the linear fit of Δj/2 versus scan rate was taken as C_dl_. The constant-current response of FeCo60-A was evaluated for 24 h at cathodic current densities of −100, −500, and −1000 mA cm^−2^. Galvanostatic chronopotentiometry was performed without iR compensation. SEM images were acquired before and after the stability test at low and high magnification. Data visualization was performed using Origin 2018 (OriginLab Corporation, Northampton, MA, USA).

## Data Availability

The raw data supporting the conclusions of this article will be made available by the authors on request.

## Supplementary Materials

The following supporting information can be downloaded at: https://www.mdpi.com/article/10.3390/gels12100932/s1. Figure S1: Textural properties of Fe-Co metal aerogels. (a–d) N2 adsorption-desorption isotherms and BET specific surface areas. (e) Pore size distributions. (f) Total pore volume and average pore diameter; Figure S2: X-ray photoelectron spectroscopy (XPS) analysis of FeCo60-A-coated carbon paper electrodes before and after HER stability testing. High-resolution XPS spectra of C 1s, O 1s, Fe 2p, and Co 2p before (top) and after (bottom) the 24 h HER stability test at −1000 mA cm^−2^; Table S1: Capacitive currents at different scan rates and calculated Cdl values for Fe-A, FeCo60-A, FeCo80-A, and Co-A; Table S2: Literature comparison of representative non-precious transition-metal-based HER electrocatalysts in 1 M KOH at high current densities [55,56,57]; Table S3: EIS fitting parameters obtained from the Nyquist spectra; Table S4: Nominal and ICP-OES-determined elemental compositions of Fe–Co aerogels; Table S5: ICP-OES analysis of Fe and Co concentrations in the electrolyte before and after HER stability testing.
