# Supplementary material for: Composition-Driven Structural Regulation of Fe-Co Aerogels for High-Current-Density Hydrogen Evolution
Source: Gels. 2026 Oct 16;12(10):932. doi: 10.3390/gels12100932 (PMC13704690; doi:10.3390/gels12100932)
Supplement: Supplementary file 1 [file gels-12-00932-s001.zip › gels-4577612-supplementary.pdf]

Supporting Information

Gels

Composition-Driven Structural Regulation of Fe-Co Aerogels for High-Current-Density Hydrogen Evolution

Sang-Hyun Kim[1], Haryeong Choi[1], Younghun Kim[2], Taehee Kim[3], and Hyung-Ho Park[1,4]**

[1] Department of Materials Science and Engineering, Yonsei University, 50 Yonsei-ro, Seodaemun-gu, Seoul 03722, Republic of Korea; sanghyun_kim@yonsei.ac.kr (S.-H.K.), haryeong@yonsei.ac.kr (H.C.),

[2] Center for Hydrogen and Fuel Cells, Korea Institute of Science and Technology (KIST), 5 Hwarang-ro 14-gil, Seongbuk-gu, Seoul 02792, Republic of Korea; younghun_kim@kist.re.kr

[3] Department of Materials Science and Engineering, Dankook University, 119 Dandae-ro, Dongnam-gu, Cheonan 31116, Republic of Korea

[4] Aerogel Materials Research Center, Yonsei University, 50 Yonsei-ro, Seodaemun-gu, Seoul 03722, Republic of Korea

***Correspondence**: taeheekim@dankook.ac.kr (T.K.); hhpark@yonsei.ac.kr (H.-H.P.)

Table of Contents

Section 1. Supplementary Tables

Section 2. Supplementary Figures

Section 1. Supplementary Tables

Table S1. Capacitive currents at different scan rates and calculated C_{dl} values for Fe-A, FeCo60-A, FeCo80-A, and Co-A.

Table S2. Literature comparison of representative non-precious transition-metal-based HER electrocatalysts in 1 M KOH at high current densities.

Table S3. EIS fitting parameters obtained from the Nyquist spectra.

Table S4. Nominal and ICP-OES-determined elemental compositions of Fe–Co aerogels.

Table S5. ICP-OES analysis of Fe and Co concentrations in the electrolyte before and after HER stability testing.

Table S1. Capacitive currents at different scan rates and calculated C_{dl} values for Fe-A, FeCo60-A, FeCo80-A, and Co-A.

The data were obtained from CV curves recorded at scan rates of 20–100 mV s^{-1} in 1 M KOH. Each current value represents half the difference between the forward and reverse scan currents at the same potential ($\Delta I/2$, mA). C_{dl} was calculated from the slope of the linear fit of $\Delta I/2$ versus scan rate.

**Scan rate (mV/s)**	**Fe-A**	**FeCo60-A**	**FeCo80-A**	**Co-A**
20	0.03314	0.03304	0.02087	0.01696
40	0.05349	0.05545	0.03855	0.0315
60	0.07847	0.07636	0.05573	0.04429
80	0.09596	0.09694	0.07254	0.0564
100	0.11764	0.11636	0.0877	0.06744
**Slope (F cm^{-2})**	0.00105735	0.00104065	0.00083825	0.00062930
**Intercept (mA cm^{-2})**	0.012299	0.013191	0.004783	0.005560
**C_{dl} (mF cm^{-2})**	1.06	1.04	0.84	0.63
**R^2**	0.9978	0.9993	0.9992	0.9973

Table S2. Literature comparison of representative non-precious transition-metal-based HER electrocatalysts in 1 M KOH at high current densities.

Catalyst & Electrode	Electrolyte	$\eta_{1,000}$ (mV)	HER Tafel Slope (mV dec^{-1})	HER Stability Test	Ref.
N-MoO_2/Ni_3S_2 NF	1 M KOH	517	76	-500 mA cm^{-2}, 10 h	[1]
WP-W_2C/W	1 M KOH	560	42.11	-1,000 mA cm^{-2}, 60 h	[2]
FeCo60-A/CP	1 M KOH	685	115	-1,000 mA cm^{-2}, 24 h	**This work**
Co-Ni_3S_2/NF	1 M KOH	850	-	-10 mA cm^{-2}, 30 h	[3]

Table S3. EIS fitting parameters obtained from the Nyquist spectra.

EIS measurements were performed over a frequency range of 100 kHz to 0.1 Hz. The spectra exhibited two partially overlapping relaxation processes and were therefore fitted using an R_s–(R_{HF} ‖ Q_{HF})–(R_{LF} ‖ Q_{LF}) equivalent circuit. The apparent R_s values were estimated from the high-frequency end of the measured spectra, while R_{LF} is reported as the apparent charge-transfer-related resistance.

**Sample**	**Apparent $R_{s,HF}$ (Ω)**	**R_{HF} (Ω)**	**Apparent R_{ct} (R_{LF}) (Ω)**	**SE of R_{LF} (Ω)**	**RMSE (Ω)**
Fe-A	1.896	3.527	1.561	0.125	0.0359
FeCo60-A	1.114	1.246	0.474	0.008	0.0135
FeCo80-A	1.781	1.605	0.877	0.053	0.0202
Co-A	1.406	1.469	0.720	0.011	0.0147

Abbreviations

R_s: series resistance (primarily solution and contact resistance);

HF: high frequency;

LF: low frequency;

R_{HF}: resistance associated with the high-frequency process;

R_{ct}: charge-transfer resistance;

R_{LF}: resistance associated with the low-frequency process;

SE: standard error;

RMSE: root-mean-square error;

Q: constant-phase-element (CPE) parameter.

Table S4. Nominal and ICP-OES-determined elemental compositions of Fe–Co aerogels.

The Fe and Co contents were determined by inductively coupled plasma optical emission spectroscopy (ICP-OES). The measured atomic ratios were calculated from the elemental mass fractions and normalized to the total Fe and Co content.

**Sample**	**Nominal Fe:Co (at%)**	**Fe (wt%)**	**Co (wt%)**	**Measured Fe:Co (at%)**
Fe-A	100:0	97.55	0.00	100:0
FeCo60-A	40:60	31.25	52.79	38.44:61.56
FeCo80-A	20:80	14.56	66.93	18.67:81.33
Co-A	0:100	0.00	87.03	0:100

Table S5. ICP-OES analysis of Fe and Co concentrations in the electrolyte before and after HER stability testing.

The Fe and Co concentrations in the electrolyte were determined by inductively coupled plasma optical emission spectroscopy (ICP-OES) to evaluate potential metal leaching during HER stability testing.

**Electrolyte sample**	**Fe**	**Co**
	**Concentration (ppm)**	**Concentration (ppm)**
Before HER test	Not detected	Not detected
After HER test	Not detected	Not detected

Section 2. Supplementary Figures

Figure S1. Textural properties of Fe-Co metal aerogels. (a–d) N_2 adsorption-desorption isotherms and BET specific surface areas. (e) Pore size distributions. (f) Total pore volume and average pore diameter.

Figure S2. X-ray photoelectron spectroscopy (XPS) analysis of FeCo60-A-coated carbon paper electrodes before and after HER stability testing. High-resolution XPS spectra of C 1s, O 1s, Fe 2p, and Co 2p before (top) and after (bottom) the 24 h HER stability test at −1,000 mA cm^{-2}.

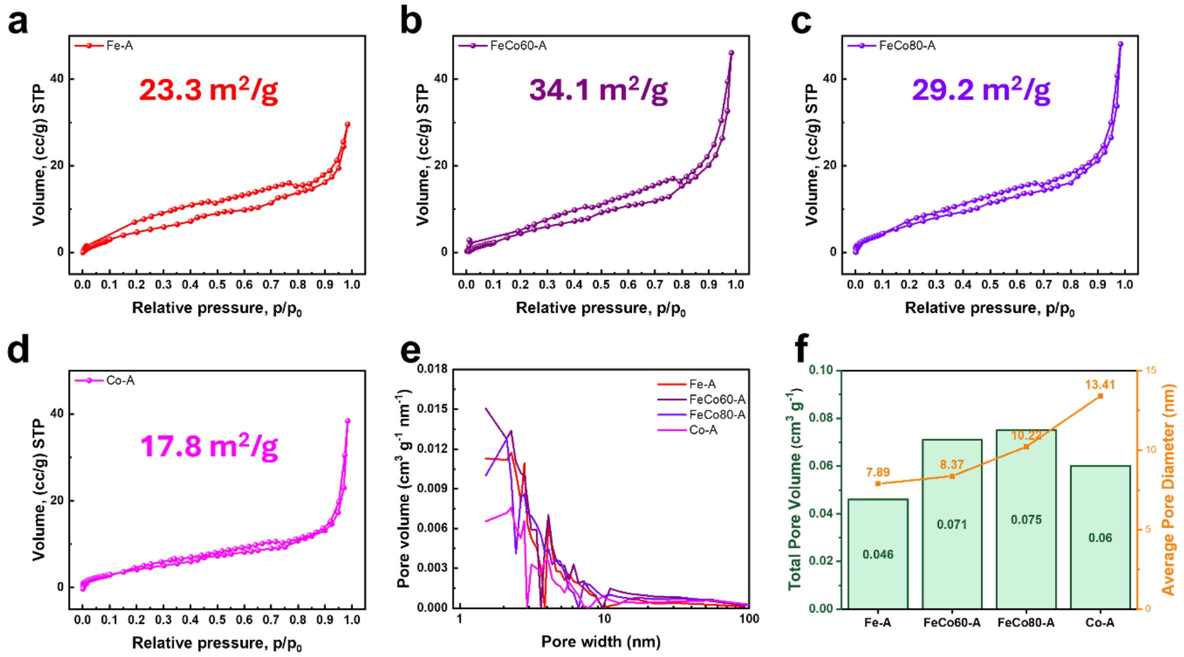

Figure S1. Textural properties of Fe-Co metal aerogels. (a–d) N_2 adsorption-desorption isotherms and BET specific surface areas. (e) Pore size distributions. (f) Total pore volume and average pore diameter.

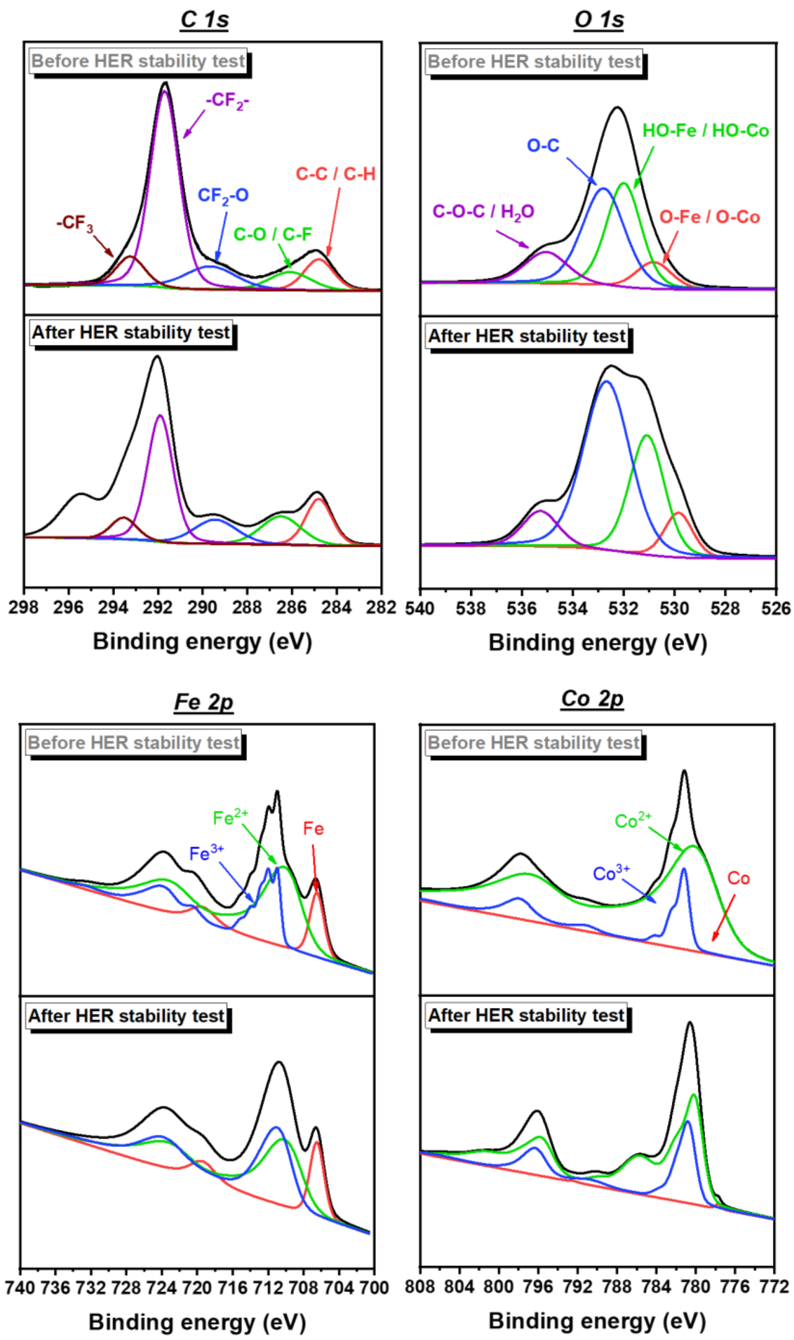

Figure S2. X-ray photoelectron spectroscopy (XPS) analysis of FeCo60-A-coated carbon paper electrodes before and after HER stability testing. High-resolution XPS spectra of C 1s, O 1s, Fe 2p, and Co 2p before (top) and after (bottom) the 24 h HER stability test at −1,000 mA cm^{-2}.

References

1. Wang, L.; Cao, J.; Lei, C.; Dai, Q.; Yang, B.; Li, Z.; Zhang, X.; Yuan, C.; Lei, L.; Hou, Y. Strongly Coupled 3D N-Doped MoO(2)/Ni(3)S(2) Hybrid for High Current Density Hydrogen Evolution Electrocatalysis and Biomass Upgrading. *ACS Appl Mater Interfaces* **2019**, *11*, 27743–27750, doi:10.1021/acsami.9b06502.
2. Huang, J.; Jian, C.; Cai, Q.; Hong, W.; Liu, W. A large scale self-supported WP–W2C nanoporous network for efficient hydrogen evolution reaction in alkaline media. *Journal of Materials Chemistry A* **2022**, *10*, 10990–10997, doi:10.1039/d2ta01307j.
3. Jian, J.; Yuan, L.; Li, H.; Liu, H.; Zhang, X.; Sun, X.; Yuan, H.; Feng, S. Hydrothermal Synthesized Co-Ni3S2 Ultrathin Nanosheets for Efficient and Enhanced Overall Water Splitting. *Chemical Research in Chinese Universities* **2019**, *35*, 179–185, doi:10.1007/s40242-019-8344-x.
